# Variant Enrichment Analysis to Explore Pathways Functionality in Complex Autoinflammatory Skin Disorders through Whole Exome Sequencing Analysis

**DOI:** 10.3390/ijms23042278

**Published:** 2022-02-18

**Authors:** Lucas André Cavalcanti Brandão, Ronald Rodrigues de Moura, Angelo Valerio Marzano, Chiara Moltrasio, Paola Maura Tricarico, Sergio Crovella

**Affiliations:** 1 Department of Advanced Diagnostics, Institute for Maternal and Child Health, IRCCS “Burlo Garofolo”, 34137 Trieste, Italy; lucabrand@gmail.com (L.A.C.B.); tricaricopa@gmail.com (P.M.T.); 2 Dermatology Unit, Fondazione IRCCS Ca’ Granda Ospedale Maggiore Policlinico, 20122 Milan, Italy; avmarzano@gmail.com (A.V.M.); moltrasiochiara@gmail.com (C.M.); 3 Department of Physiopathology and Transplantation, Università degli Studi di Milano, 20122 Milan, Italy; 4 Department of Medical Surgical and Health Sciences, University of Trieste, 34137 Trieste, Italy; 5 Biological Science Program, Department of Biological and Environmental Sciences, College of Arts and Sciences, University of Qatar, Doha 2713, Qatar; sgrovella@qu.edu.qa

**Keywords:** whole exome sequencing, OMICs, hidradenitis suppurativa, PASH, PAPASH, SAPHO

## Abstract

The challenge of unravelling the molecular basis of multifactorial disorders nowadays cannot rely just on association studies searching for potential causative variants shared by groups of patients and not present in healthy individuals; indeed, association studies have as a main limitation the lack of information on the interactions between the disease-causing variants. Thus, new genomic analysis tools focusing on disrupted pathways rather than associated gene variants are required to better understand the complexity of a disease. Therefore, we developed the Variant Enrichment Analysis (VEA) workflow, a tool applicable for whole exome sequencing data, able to find differences between the numbers of genetic variants in a given pathway in comparison with a reference dataset. In this study, we applied VEA to discover novel pathways altered in patients with complex autoinflammatory skin disorders, namely PASH (*n* = 9), 3 of whom are overlapping with SAPHO) and PAPASH (*n* = 3). With this approach we have been able to identify pathways related to neutrophil and endothelial cells homeostasis/activations, as disrupted in our patients. We hypothesized that unregulated neutrophil transendothelial migration could elicit increased neutrophil infiltration and tissue damage. Based on our findings, VEA, in our experimental dataset, allowed us to predict novel pathways impaired in subjects with autoinflammatory skin disorders.

## 1. Introduction

Since the introduction of Next Generation Sequencing, the quantity of molecular biological information associated with diseases has started rising, reaching so a great volume that the term “big data” has been applied to these findings [1].

We are in the post-genomic era: genomic information has become easily available, and useful for helping researchers and clinicians to better understand the biological processes occurring in human diseases through the analysis of multiple information sources [2].

Genomic studies have contributed to unravelling, at least in part, the molecular bases of some disorders, prompting the discovery of molecular targets (biomarkers), usually associated with disease’s onset, prognosis, and treatment [2]. Nonetheless, several genomic findings, even if already published/available, have not been integrated in a broader biological sense, but have only been considered individually in a unique clinical context; as an example, large Genome Wide Association Studies (GWAS) projects have identified novel genetic variants associated with pathological conditions without successfully leading to a full understanding of the pathogenic mechanisms of the disease [3].

Therefore, the extensive explanation of a disorder based only on association studies looking for common variants could fail due to the high statistical pressure occurring when performing a GWAS. Thus, new approaches should be investigated to unravel and better understand the complexity of a disease. Currently, several questions have arisen on this topic: How many and what type of studies are necessary to disclose the role of the genome in the pathogenesis of a disease? What is the information that can be retrieved by genomic studies? How can we integrate the genomic information in favor of an improved and clear etio-pathologic scenario? Several scientific responses regarding diseases’ etio-pathogenesis, in which genetics is supposed to play a role, have already been produced, but have not been interpreted at a coherent biological level, since they have not been analyzed in an integrated manner, having only been considered individually.

In a previous work, we suggested a novel Whole Exome Sequencing (WES) approach to identify the biological pathways commonly disrupted in syndromic HS patients, by focusing on deleterious variants spread on a patient’s genome [4].

In this study, we develop a new workflow, called Variant Enrichment Analysis (VEA), to determine which human pathway carries the most genetic variants in order to predict the pathway’s impairment. We describe how to use WES findings not only to search for genetic variations associated with a disease, but to investigate the molecular mechanisms and etio-pathogenic pathways involved in its onset.

VEA was used to interpret the WES findings of 12 unrelated patients affected by complex autoinflammatory skin disorders, including PASH (pyoderma gangrenosum, acne, and hidradenitis suppurativa), PAPASH (pyogenic arthritis, pyoderma gangrenosum, acne, and hidradenitis suppurativa), and SAPHO (synovitis, acne, pustulosis, hyperostosis, osteitis), another autoinflammatory disease that may share cutaneous manifestations with PASH.

PASH, PAPASH, and PASH/SAPHO overlapping syndrome are diseases belonging to a single clinicopathological spectrum having abnormal innate immunity activation as a major pathogenetic driver; patients suffering from these complex diseases and clinically characterized by multifaceted clinical phenotypes [5] (see Materials and Methods for patients’ clinical descriptions) have been investigated in recent years for the presence of individual specific genetic variations [6,7]. Despite having identified novel mutations associated with PASH, PAPASH, etc., the molecular mechanisms at the basis of these autoinflammatory skin disorders have not been fully elucidated, thus limiting the relevance of genetic analysis in the discovery of patients’ unique individual mutations. Therefore, we used VEA to analyze WES data from unrelated PASH, PAPASH, and PASH/SAPHO patients with the aim of discovering novel pathways characterizing the different clinical phenotypes.

## 2. Results

The WES data obtained on PASH, PAPASH, and PASH/SAPHO patients, including genetic variation information as well as pathway description, are detailed at https://davinci.biohub.solutions/pash/ (Figures S1–S7 and Tables S1–S10). All the information about the VEA pipeline, as well as the statistical methods, is reported in Section 4 of this article.

Here, we will describe our WES analysis approach focusing on VEA findings for the three patient groups. Firstly, we were able to determine which pathways carry greater numbers of genetic variants in comparison to the general population by using the False Discovery Rate (FDR) method. Then, on the basis of a Venn diagram, we identified exclusive enriched pathways (eEP) between the studied groups.

### 2.1. Exclusive Enriched Pathways (EEP)

To determine the enriched pathways, we imputed only the common variants shared by all individuals in each patient group. We retrieved through VEA 33, 40 and 28 enriched pathways (EP) in the PAPASH, PASH, and PASH/SAPHO patient groups, respectively.

In the Figure S7 (https://davinci.biohub.solutions/pash/data/data1/vea/#figure-s7-1), the exclusive EP (eEP), represented using a Venn diagram, are reported: 15, 25 and 12 specific eEP were observed in the PAPASH, PASH, and PASH/SAPHO patient groups.

### 2.2. EEP from PAPASH

Fifteen exclusive enriched pathways were found for the PAPASH group (Table S10.1, https://davinci.biohub.solutions/pash/data/data1/vea/#table-s10-1). Ranking the eEP based on the important variants (ImportantVar), we found the following impaired pathways: the endosomal/vacuolar pathway Reactome ID (R-HSA)-1236977), with the highest number of ImportantVar (*n* = 78 importantVar), and the alpha-defensins (R-HSA-1462054) and fatty acids (R-HSA-211935) pathways, both possessing 11 ImportantVar. Moreover, defective CYP11B2 causes CMO-1 deficiency (R-HSA-5579009) and Glycogen storage disease type 0 (R-HSA-3858516), which according to VEA achieved the greatest odds ratios (OR), 5.03 and 3.94, respectively.

Three eEP were retrieved on the basis of their expression probability ratio (EPR) ranking: mTORC1-mediated signaling (R-HSA-166208), noncanonical activation of NOTCH3 (R-HSA-9017802), and Neutrophil degranulation (R-HSA-6798695).

### 2.3. EEP from PASH

In the PASH patient group, we found 25 eEP. The Dectin-2 Family (R-HSA-5621480) was the eEP with the highest number of ImportantVar, reaching 200 variants distributed in 10 genes (Table S10.3 https://davinci.biohub.solutions/pash/data/data1/vea/#table-s10-2). Another four eEP were also detected according to their ImportantVar ranking: Termination of O-glycan biosynthesis (R-HSA-977068), Defective GALNT12 causes colorectal cancer 1 (CRCS1) (R-HSA-5083636), Defective GALNT3 causes familial hyperphosphatemic tumoral calcinosis (HFTC) (R-HSA-5083625), and Defective C1GALT1C1 causes Tn polyagglutination syndrome (TNPS) (R-HSA-5083632). These four eEP are involved in the biosynthesis of O-glycan oligosaccharides and share genes with the Dectin-2 Family pathway. In addition, Downstream TCR signaling (R-HSA-202424) and its related pathways were observed.

We then ranked the eEP according to their OR, thereby considering all eEP carrying at least one ImportantVar. Two eEP were observed: FGFR4 mutant receptor activation (R-HSA-1839128) and betaKlotho-mediated ligand binding (R-HSA-1307965). Both pathways are involved in signaling by FGFR4 pathways.

Checking the expression probability ratio (EPR) ranking, the following five pathways were detected: Release of apoptotic factors from the mitochondria (R-HSA-111457), Degradation of GLI2 by the proteasome (R-HSA-5610783), Cross-presentation of soluble exogenous antigens (endosomes) (R-HSA-1236978), Synthesis of PI (R-HSA-1483226), and Cell surface interactions at the vascular wall (R-HSA-202733).

### 2.4. EEP from PASH/SAPHO

Twelve eEP were identified in the PASH/SAPHO patient group (Table S10.3 https://davinci.biohub.solutions/pash/data/data1/vea/#table-s10-3). Then, we ranked the eEP according to ImportantVar, OR, and expression probability ratio (EPR). Signaling by Rho GTPases (R-HSA-194315), Macroautophagy (R-HSA-1632852) and Crosslinking of collagen fibrils (R-HSA-2243919) possessed the highest numbers of ImportantVar. Four eEP were found by OR ranking: Invadopodia formation pathway (R-HSA-8941237), SDK interactions (R-HSA-373756), Canonical retinoid cycle in rods (R-has-2453902), and Josephin domain DUBs (R-HSA-5689877).

Five eEP were revealed by EPR: Josephin domain DUBs (R-HSA-5689877), XBP1(S) activates chaperone genes (R-HSA-381038), Macroautophagy (R-HSA-1632852), Mitochondrial tRNA aminoacylation (R-HSA-379726), and Collagen formation (R-HSA-1474290).

## 3. Discussion

Whole exome analysis from a single individual produces thousands of pieces of information; this large data set represents all the puzzle’s pieces of a patient’s results, as in the case of genetic variant analysis in unrelated patients sharing similar clinical phenotypes [8,9].

In molecular pathology, the molecular pathway represents an advanced hierarchical level that makes it possible to describe molecular integration, cell coordination, and the function of tissues and organs in a disease; therefore, the ability to translate exome variant data into predictions of pathway alterations could be useful for mechanically deciphering diseases, especially in cases where only a patient’s DNA is available, as in our study.

Therefore, with the aim of increasing the amount of biologically useful information retrievable from WES, we developed a new workflow, called variant enrichment analysis (VEA), to identify which pathways carry the largest numbers of genetic variants (ImportantVar), thereby predicting pathway impairment. Starting from whole exome data of 12 PAPASH, PASH, and PASH/SAPHO patients, VEA was able to integrate variants distributed in the human exome by assembling them into hierarchical patterns. VEA was designed to investigate clinically related issues when low numbers of individuals are available, as in our case. Our new approach does not need to rely on high statistical power to obtain results that provide useful insights into the pathways disrupted in a disease. Other genetic approaches, like GWAS, are characterized by relevant statistical pressures, and need to achieve high statistical power to produce interpretable results; therefore, they are not able to exploit the correlation between the number of SNPs in a particular gene and the related molecular pathway in situations where a small number of individuals is available. Notably, VEA was developed to analyze small numbers of individuals.

### VEA Applied to Inflammatory Skin Disorders: PAPASH, PASH, and PASH/SAPHO

Hidradenitis suppurativa is a common feature of PASH, PAPASH, and PASH/SAPHO; these syndromes are elicited by a sterile and recurrent autoinflammation leading to several dermatological manifestations [10]. It is a well-known fact that the three conditions share an ethological pattern, including the molecular signature of IL-1b and TNF-a [10]; however other key molecular players could be involved in these disorders. Our VEA analysis allowed us to identify novel molecular pathways characterizing PASH, PAPASH, and PASH/SAPHO syndromes.

**Figure 1:**
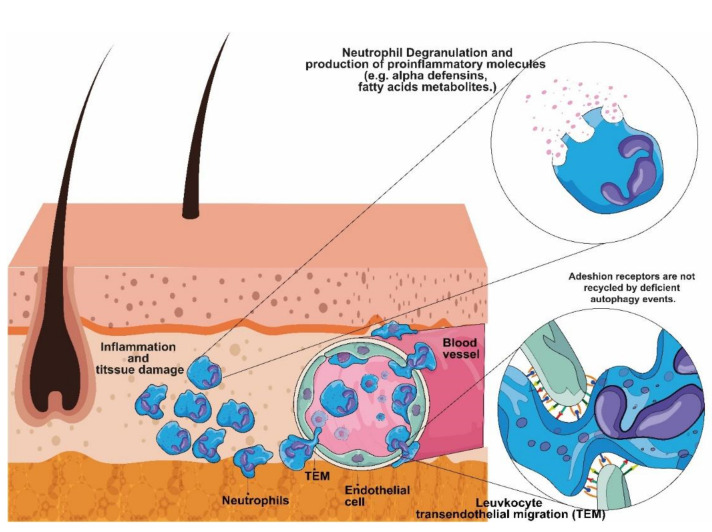
Exploiting Variant Enrichment Analysis (VEA) results on the basis of molecular pathogenesis of PAPASH, PASH, and PASH/SAPHO. The figure represents the unregulated mechanisms of leukocyte transendothelial migration (TEM). VEA revealed that the pathways involved in neutrophil infiltration and activation are altered in PAPASH, PASH, and PASH/SAPHO patients.

In Figure 1, we describe the pathways, as determined by VEA, involved in the three disorders.

The exclusive enriched pathways (eEP) found in PAPASH patients showed that two of them were related to autophagy: the endosomal/vacuolar pathway (R-HSA-1236977) and the mTORC1-mediated signaling pathway (R-HSA-166208). PASH/SAPHO patients showed impairment in the macroautophagy pathway (R-HSA-1632852).

Autophagy is a crucial event responsible for maintaining cellular homeostasis, including keratinocytes and endothelial cells (EC) [11]. In fact, unregulated autophagy is involved in epidermal differentiation defects, and it has also been shown to be involved in dermatological diseases [12,13]. Moreover, autophagy deficiency in EC results in unregulated leukocyte transendothelial migration, increasing neutrophil infiltration and tissue damage [14].

Moreover, our results showed that PASH patients showed alterations in the cell surface interactions at the vascular wall (R-HSA-202733) pathway. According to Reglero-Real et al. (2021) [14], autophagy regulates leukocyte transendothelial migration by recycling the molecular adhesion receptors on the cell surfaces of EC, thus indicating that PASH patients could also experience increased neutrophil infiltration and tissue damage. In fact, neutrophils have been reported to be involved in skin inflammation of neutrophilic dermatoses (ND), including Pyoderma Grangrenosum (PG), present in the conditions PAPASH and PASH [15]. Neutrophil degranulation, alpha-defensins and noncanonical activation of NOTCH3 also play a role in PAPASH. All of the pathways described above could be connected to the neutrophil inflammatory response [16] (Figure 1).

SAPHO is a rare disorder presenting clinical manifestations different from PAPASH and PASH. The main phenotype of SAPHO is bone hyperostosis caused by sterile osteitis and osteomyelitis. Interestingly, three enriched exclusive pathways (eEP) found in SAPHO patients were related to extracellular matrix organization (R-HSA-8941237, R-HSA-2243919 and R-HSA-1474290), suggesting their possible involvement in SAPHO pathogenesis.

## 4. Material and Methods

### 4.1. Patients

Twelve Italian patients with autoinflammatory skin disorders, recruited between 2011 and 2021 and followed-up at the Dermatology Unit, Fondazione IRCCS Ca’ Granda Ospedale Maggiore Policlinico, Milan, Italy, were enrolled in this study: six men and six women with median age of 38.67 years (17–61). All patients answered a standard questionnaire and signed written informed consent approved by the Area B Milan Ethics Committee (protocol no. 487_2020).

Nine patients were affected by PASH, three of whom had a form overlapping with SAPHO, and three were diagnosed with PAPASH. All PASH patients displayed inflammatory nodules that progressed into abscesses and draining fistulas, consistent with HS; acne; and ulcers compatible with PG. The patients with PAPASH showed the clinical triad of PASH associated with pyogenic arthritis. The disease presented a chronic/relapsing state for all cases. Family history for PG or HS was negative for all individuals. Four patients presented smoking as a risk factor, whereas obesity was considered an aggravating factor in two other cases. Relevant comorbidities were: Crohn’s disease (*n* = 2); polycystic ovary syndrome (*n* = 4); pilonidal cyst (*n* = 1); psoriasis (*n* = 1); type II diabetes mellitus (*n* = 1); and ulcerative colitis (*n* = 1). The patient with type II diabetes developed a gliptin-induced bullous pemphigoid. Regarding the affected regions, axillae (*n* = 12) was the most frequent area, followed by groin (*n* = 10), anogenital region (*n* = 8), nuchal region (*n* = 4), intergluteal folds (*n* = 4) and submammary/intermammary folds (*n* = 4). The average International Hidradenitis Suppurativa Severity Score System (IHS4) score was 22.11.

The PG phenotype was considered as ulcerative in all patients and was associated with the vegetating aspect in three cases. Age at PG onset was generally concomitant with HS onset, with an average difference of 3.7 years between the two diseases. The most recurrent sites involved were the lower limbs (*n* = 6), trunk (*n* = 4), upper limbs (*n* = 2), peristomal site (*n* = 2) and perianal region (*n* = 1). PG was localized in four cases, multilesional in six cases, and widespread in two cases.

Acne was present in the enrolled subjects, with the face (in all patients) and trunk (in six patients) being the most-affected areas. The average global acne grading system (GAGS) score was 26.78. Acne onset happened before the appearance of HS or PG lesions in all patients, with an average time between acne onset and development of PG/HS of 8 years. Arthritis in the wrists and ankles was observed in three cases with PAPASH, whereas the three patients with overlapping PASH/SAPHO showed sacroiliac joint, ankle, and spine involvement. The average age at onset of arthritis was 25.67 years.

All cases were treated with topical therapy, including clindamycin gel for HS lesions. Systemic antibiotics were given to ten patients, while oral retinoids (isotretinoin) were administered to one individual. Five patients received immunosuppressive/immunomodulating agents, those being: systemic corticosteroids (*n* = 3); cyclosporine (*n* = 2); dapsone (*n* = 2); IVIG (*n* = 1); methotrexate (*n* = 1); and colchicine (*n* = 1). Monoclonal antibodies were administered to all patients: adalimumab (*n* = 6); infliximab (*n* = 6) andustekinumab (*n* = 2). Anakinra was also administered to four individuals. There was no need for surgical intervention for any of the enrolled patients.

### 4.2. DNA Extraction and Exome Sequencing

Genomic DNAs was extracted from saliva samples using the Oragene-DNA (Oragene^®^, Ottawa, ON, Canada) kit following the manufacturer’s instructions. Agarose gel (2%) and Qubit instrument (Invitrogen^®^, OR, USA) were used to evaluate DNA quantity and quality prior to sequencing.

Exome sequencing was outsourced and performed by Macrogen Europe (Amsterdam, The Netherlands). The Exome Sequencing Analysis, with a declared 150× coverage, used the Illumina^®^ SureSelect Human V7 Kit Library preparation and sequencing reaction, in an Illumina^®^ HiSeq 2500 System, generating pair-end reads of 150 base pairs.

### 4.3. Exome Annotation and Global Analysis

The exome annotation and data analysis workflow consisted of five steps:

(1) Quality control of the fastq.gz input is performed before the procedures using fastQC (https://www.bioinformatics.babraham.ac.uk/projects/fastqc/, accessed on 31 October 2021), with which an overall summary of the sequencing performance can be assessed (e.g., total sequences, sequence length, GC proportions, sequence quality score, and adapter content); after that, library adapters and reads (single or pair-ended) with lengths below 25 base pairs and with low Phred score (Q < 20) are removed using the Trim Galore application (http://www.bioinformatics.babraham.ac.uk/projects/trim_galore/, accessed on 31 October 2021).
(2) The polished.fastq.gz files with the raw reads after QC are aligned using the Burrows-Wheeler Aligner software package [17], mapping them using the most recent Reference Human Genome version (GRCh.38).
(3) Picard tools (https://broadinstitute.github.io/picard/, accessed on 31 October 2021) are used for marking and remove duplicate reads, and GATK (https://software.broadinstitute.org/gatk/, accessed on 31 October 2021) is used for base recalibration. During the variant calling step, Strelka2 is used for variant calling [18]. The Manta software can also be used, before Strelka2, when sequencing is being performed with the aim of finding somatic mutations [19]. GATK and vcftools [20] are used again to exclude low-quality variants.
(4) Variant annotation is performed using ANNOVAR [21] software, using databases related to the GRCh.38 reference genome. R Software [22] is used to manipulate the ANNOVAR results for the purposes of descriptive and inferential analysis.
(5) The analysis has two main parts: individual analysis, which consists of summarizing the descriptive data for each sequenced sample, and group data analysis, which aims to draw comparisons between groups of patients. Additionally, for both the individual and group categories, the so-called ‘Variant Enrichment Analysis’ is performed.

The fastaq is available at SRA: https://www.ncbi.nlm.nih.gov/sra/PRJNA801118.

### 4.4. Variant Enrichment Analysis (VEA)

Variant Enrichment Analysis (VEA) is based on Pathway Enrichment Analysis for expression data [23]. We searched for statistical differences between the number of genetic variants in each pathway and that in a reference dataset (background). We used the R package ‘ReactomePA’ and ‘reactome.db’ in order to fetch pathway information of each gene containing at least one variant in the dataset [23]. The Reactome Pathway database is also available at https://reactome.org/PathwayBrowser/ (accessed on 31 October 2021), where each human pathway entry receives the R-HSA pattern identification.

The reference dataset was obtained from GnomAD Exome v3.0 [24]. As the occurrence of some genetic variants may be related to specific population genetic background, we considered only the Non-Finnish European (nfe) common variant information in the reference dataset, since the case population included in this study is from Italy. Common variants were defined as variants presented in all individuals from a given dataset.

Once we had the number of common variants per pathway from the case population and reference dataset, we used Fisher’s Exact test with False Discovery Rate (FDR) to identify statistical differences in the proportion of variants. Adjusted *p*-values < 0.05 were considered as significant in this analysis. All analyses were performed using the R software package (https://www.r-project.org, accessed on 31 October 2021).

**Figure 2:**
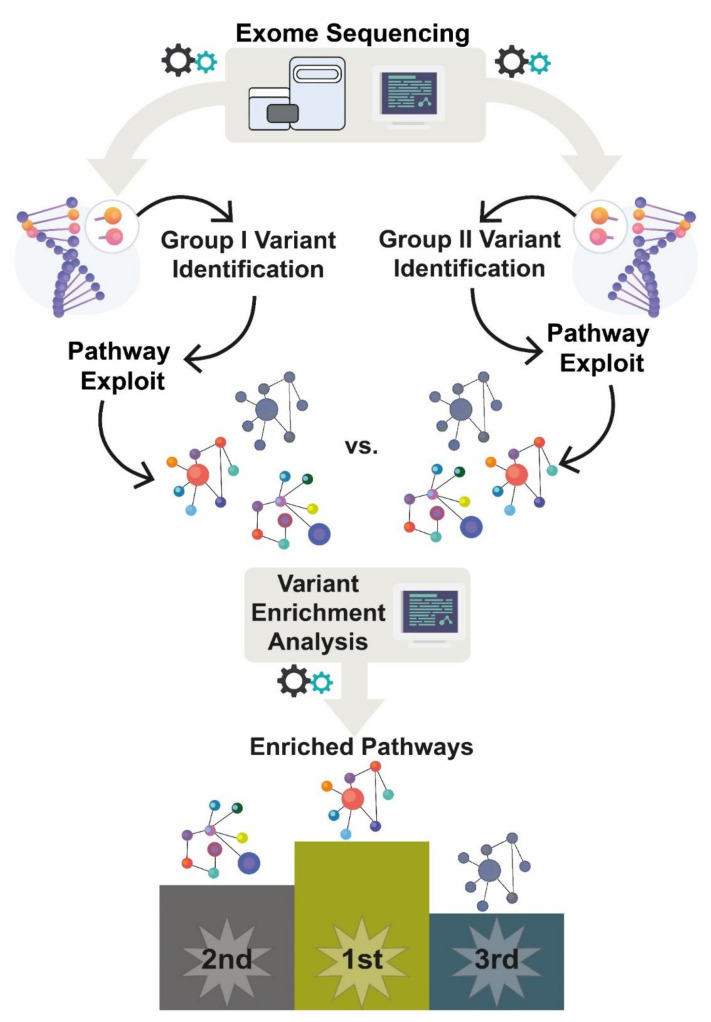
Variant Enrichment Analysis. After sequencing all patients, we extracted common variants (CommonVar) from each studied group, PAPASH, PASH, and PASH/SAPHO. Then, we recovered all genes carrying CommonVar and performed a VEA. Finally, a Venn diagram (Figure S7: https://davinci.biohub.solutions/pash/data/data1/vea/#figure-s7-1) was used to set apart the exclusive enriched pathway (eEP) for each group.

The initial list of Enriched Pathways was filtered through a Venn diagram to set apart exclusive enriched pathways (eEP) for each studied group (PASH, PAPASH, and overlapping PASH/SAPHO). Then, we ranked the eEP based on odds ratio (OR) higher than 1.5 and by the number of important variations (ImportantVar) presented in each eEP. ImportantVar is defined as genetic variants with a positive damage prediction score, based on multiple algorithm prediction (CADD, SIFT, PolyPhen2, FATHMM, etc), and variants with functional impact, such as non-synonymous, stop codon, and start codon variations. The VEA approach is schematically presented in Figure 2.

To determine whether one of the eEP was expressed on normal or inflammatory lesional skin, and thus, involved in the pathogenesis of PASH, PAPASH, and PASH/SAPHO, we considered gene and protein expression. Since skin biopsies from the enrolled patients were not available, we searched public databases of gene and protein expression to determine the ratio of protein and mRNA expression on normal or inflammatory lesional skin. We traced back all genes/proteins from a given eEP to identify the expression profile in normal or inflamed skin. Normal skin expression (protein and mRNA) was recovered from the Human Protein Atlas database [25]. We calculated the expression probability ratio (EPR) for a given pathway in skin, dividing the number of genes/proteins expressed per total genes/proteins for the given pathway.

Data expression for lesional skin was restricted only to mRNA expression, as referenced from studies performed by Hoffman et al. (20018) [26] and Penno et al. (2020) [27] on skin biopsies of HS patients. The differentially expressed genes (DEGs) between lesional and perilesional skin were used as input to perform enrichment pathway analysis [23]. As a last step, a Venn diagram was employed, including the enriched pathways achieved by DEGs list and the exclusive enriched pathway (eEP) of each patient group.

## 5. Conclusions

Variant Enrichment Analysis was developed on the basis of Pathway Enrichment Analysis from ReactomePA [23]. Two pieces of information are required to perform VEA: the list of variants found in exome sequencing; and reference population data, used as a background dataset. Thus, the VEA workflow can be easily implemented to picture pathway impairment in an individual exome or in a group of individuals. Our VEA approach is applicable in situations of individual reports or in cases with low numbers of subjects, where the statistical power of genetic findings, in the case of GWAS, fails.

Indeed, VEA allowed us to identify specific pathways involved in PASH, PAPASH, and PASH/SAPHO pathogenesis. PASH, PAPASH, and PASH/SAPHO are rare and complex conditions, where single nucleotide variants could fail to contribute to unraveling the etio-pathogenesis of the diseases.

Due the lack of skin patient transcriptome, we also coupled VEA with an in silico expression evaluation, based on expression database resources, in order to better support our findings, thus providing an incremental layer of VEA interpretation.

Recently, we launched a multi-omics platform (PlatOMICs), an integrative web platform for analyzing omics (or multi-omics) data, to unravel the molecular pathogenesis of human disorders [28]. Unfortunately, since no skin biopsy from our patients was available, we could not apply this integrative analysis. We are also aware of the main limitation of our study, whereby we did not compare our findings with other omics data, in particular, transcriptomics and proteomics from the skin.

Finally, we note that the novel VEA strategy based only on WES data contributes to changing the vision of genetic analysis and its objectives (i.e., finding genetic variations associated with certain diseases), thus representing a modern tool for translating exome variants into a pathological context.

## Data Availability

Supporting data can be accessed at https://davinci.biohub.solutions/pash/.

## Supplementary Materials

The following supporting information can be downloaded at: https://davinci.biohub.solutions/pash/.
